# Scaling up discovery of hidden diversity in fungi: impacts of barcoding approaches

**DOI:** 10.1098/rstb.2015.0336

**Published:** 2016-09-05

**Authors:** Rebecca Yahr, Conrad L. Schoch, Bryn T. M. Dentinger

**Affiliations:** 1 Royal Botanic Garden Edinburgh, 20A Inverleith Row, Edinburgh, UK; 2 National Center for Biotechnology Information, National Library of Medicine, National Institutes of Health, Bethesda, MD, USA; 3 Royal Botanic Gardens Kew, Richmond, Surrey, UK; 4 Institute of Biological, Environmental and Rural Sciences, Aberystwyth University, Cledwyn Building, Penglais, Aberystwyth SY23 3DD, UK

**Keywords:** internal transcribed spacer, fungi, barcoding, diversity, metabarcoding, taxonomy

## Abstract

The fungal kingdom is a hyperdiverse group of multicellular eukaryotes with profound impacts on human society and ecosystem function. The challenge of documenting and describing fungal diversity is exacerbated by their typically cryptic nature, their ability to produce seemingly unrelated morphologies from a single individual and their similarity in appearance to distantly related taxa. This multiplicity of hurdles resulted in the early adoption of DNA-based comparisons to study fungal diversity, including linking curated DNA sequence data to expertly identified voucher specimens. DNA-barcoding approaches in fungi were first applied in specimen-based studies for identification and discovery of taxonomic diversity, but are now widely deployed for community characterization based on sequencing of environmental samples. Collectively, fungal barcoding approaches have yielded important advances across biological scales and research applications, from taxonomic, ecological, industrial and health perspectives. A major outstanding issue is the growing problem of ‘sequences without names’ that are somewhat uncoupled from the traditional framework of fungal classification based on morphology and preserved specimens. This review summarizes some of the most significant impacts of fungal barcoding, its limitations, and progress towards the challenge of effective utilization of the exponentially growing volume of data gathered from high-throughput sequencing technologies.

This article is part of the themed issue ‘From DNA barcodes to biomes’.

## 1. Introduction

Diversity in the fungal kingdom is estimated to range from 1.5 to more than 5 million species [1–3], but only a small fraction of these species (approx. 100 000) have so far been described [4], despite their essential roles in ecological systems in terms of global chemical cycling, decomposition, nutrient acquisition in symbiosis and pathogenicity [5–8]. Because these eukaryotic organisms have microscopic life-history stages with simple and often convergent morphological features, genetic data are essential for quantifying the extent and distribution of their diversity. Early molecular studies focused on fungi relevant to medical and industrial applications, but within little more than a decade, surveys of the natural environment were being used to uncover hidden fungal diversity, all based on universal nuclear ribosomal primers developed by White *et al.* [9]. The development of these primers was perhaps the most important advance in establishing a barcoding approach—using standard, short sequences to identify taxa, facilitating comparative research across diverse fungal groups and ultimately becoming the standard practice.

The formal acceptance of the internal transcribed spacer (ITS) region in the nuclear ribosomal cistron as the standard fungal barcode was based on a phylogenetically wide-ranging test showing reasonable discriminatory power at the species level in many groups [10]. This built on an extensive body of literature showing that discontinuities in sequence variation often correspond to data from morphology, chemistry, biogeography and ecology [11]. Comparisons with mitochondrial cytochrome oxidase 1 (CO1), the standard barcode marker for animals, showed that in many fungi CO1 is prone to having multiple introns and is difficult to amplify with universal primers [12–14]. There is now an extensive set of resources for fungal barcoding, including sampling protocols and laboratory techniques, summarized in the electronic supplementary material.

The aim of this paper is to review how DNA barcoding has been deployed to enhance understanding of global fungal diversity, including both scientific advances and societal applications, focusing on ITS barcoding and extending to genome-wide sequencing. We build on more than 20 years of data collection using the originally de facto and now formal ITS barcode marker, and we reiterate the challenge to integrate DNA sequence data into the wider historical classification framework for fungi [15–17]. Given the scale of this challenge, and the increasingly urgent need to rationalize these two approaches, it is clear that one important role for DNA barcoding will be to generate novel species hypotheses as well as evaluating existing taxon concepts. Although unrelated lineages such as oomycetes share fungus-like lifestyles, research challenges and even barcoding target loci [18], we restrict this review to true fungi.

## 2. Fungal barcoding databases

Effective DNA barcoding requires comparing newly generated sequences to a well-established reference database. This voucher-based approach enabling reproducibility and re-examination was advocated from the early stages of fungal barcoding, with sequence data routinely accompanied by curated and annotated specimens or strains [19]. However, two important challenges have persisted. First, despite concerted efforts to fill the gap, only a small proportion of fungal species have ITS data in the public databases, such as GenBank, which form part of the International Nucleotide Sequence Database Collaboration (INSDC) [20–23]. Second is the accumulation of misidentified and unspecified sequences in public sequence databases [24,25], which make identifications using these sequences problematic.

GenBank and UNITE are the main repositories of fungal sequence data. These data are augmented by various specialized sequence databases for barcoding and barcoding-related work, which collate and curate databases of reference material (voucher specimens or cultures) linked to sequence data (table 1). In addition, several specialized bioinformatics pipelines for high-throughput fungal analyses have been devised [36–38]. The UNITE database and PlutoF [39] workbench include modules for ITS extraction, chimera checking, and identification, including matching query sequences with species hypotheses (including varying similarity cut-offs) and reference sequences determined by expert users [36]. The Ribosomal Database Project (RDP) employs a naive Bayesian approach to classify unknown sequences, relying on initially selected training sets [40], including ITS [41]. Another important initiative at the US National Center for Biotechnology Information (NCBI) is focused on curating and re-annotating ITS sequences from type material that is already publically available at the INSDC, i.e. the RefSeq Targeted Loci ITS project [32,42]. Curated databases with a guarantee of long-term support are critically important, because the community-led specialist fungal databases often lack such funding commitments.

**Table 1.** A selection of actively curated specialist databases containing ITS reference sequences for fungi, including single and multilocus sequence typing (MLST) resources.

name	scope	contents	web address, NCBI nucleotide Entrez search term(s)
CBS-KNAW: Centraalbureau voor Schimmelcultures BioloMICS Databases [26]	Aspergillus and Penicillium Dermatophytes *Fusarium* indoor fungi medical fungi *Phaeoacremonium* *Pseudallescheria/Scedosporium* Resupinate Russulales *Russula* yeasts	ITS and MLST	www.cbs.knaw.nl
Fusarium-ID [27,28]	*Fusarium*	multiple markers, ITS	http://isolate.fusariumdb.org
International Society of Human and Animal Mycology (ISHAM) ITS database [29]	human and animal pathogens	ITS	http://its.mycologylab.org loprovishamits[filter]
MaarjAM [30]	Glomeromycota	multiple markers, ITS	http://maarjam.botany.ut.ee
Q-Bank [31]	quarantine organisms	ITS and MLST	www.q-bank.eu/fungi
RefSeq Targeted Loci [32]	mainly sequences from type material, re-annotated from INSDC	ITS, LSU	http://www.ncbi.nlm.nih.gov/refseq/targetedloci/ 177353[bioproject] 51803[bioproject]
RDP Classifier [33]	wide taxonomic range, training set selected from INSDC	ITS, LSU	https://rdp.cme.msu.edu/classifier/classifier.jsp
Targeted host-associated Fungi ITS Database (THF) [34]	wide taxonomic range, re-annotated from INSDC	ITS	https://risccweb.csmc.edu/microbiome/thf/
TrichoBLAST [35]	*Trichoderma* and its *Hypocrea* synonyms	*TEF1*, *RPB2*, ITS	www.isth.info/tools/blast/index.php
UNITE [36]	wide taxonomic range, re-annotated from INSDC	ITS	http://Unite.ut.ee loprovunite[filter]

## 3. Linking names and sequences

**Figure 1.**
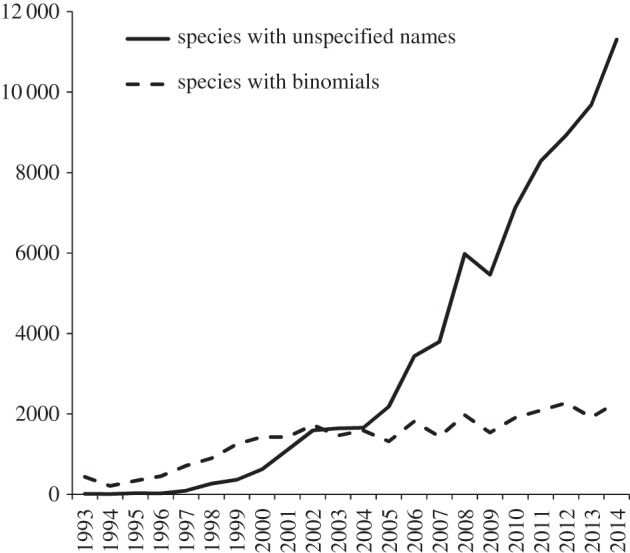
Number of fungal binomial species names and unspecified binomials added each year—‘dark taxa’ in GenBank.

**Figure 2.**
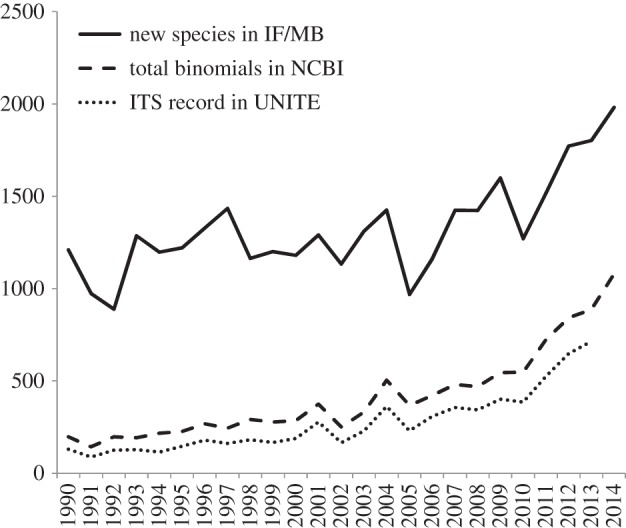
Number of new species listed in Index Fungorum by year, and their representation in the NCBI Taxonomy Database. New species names in Index Fungorum per year (solid line), their presence in NCBI Taxonomy (dashed line) and which of them have ITS sequences in the UNITE database (version 7; dotted line).

Ultimately, linking scientific names to molecular data requires reference sequences generated from type material. Recent efforts to add barcodes for cultures and specimens are beginning to make inroads into this problem [32,43]. However, the problem of ‘dark taxa’ represented by sequences lacking formal binomials is steadily growing [44,45]. The latest comparisons of the names in the NCBI Taxonomy Database [46] indicate a shift in the early 2000s where more sequences were released without, rather than with, species-level identification; this trend was recognized by Hibbett *et al*. [17] in 2011 and has not diminished (figure 1). Compounding the ‘sequences without names’ issue is the ‘names without sequences’ problem (figure 2). From a 10 year period up to 2009, more than 70% of new fungal species described had no ITS sequence deposited [17]. This in part is driven by some researchers having limited access or resources for DNA sequencing, and in part by researchers not choosing to generate the sequence data for new species. After the requirement for online deposition of new fungal names was proposed in 2011, the percentage of new species with sequences has increased to 55%. Another important improvement to NCBI, implemented in 2013, is allowing material to be retrospectively designated by curators as from a type in the taxonomy database; links can also be made directly to outside biorepositories [47]. As of 2016, 23% (7308) of current fungal species with binomials (32 431) in the INSDC databases (November 2015) can be tied to sequences from type material with 14% (4759) having quality verified ITS sequences in the UNITE database.

Massively parallel metabarcoding, the use of high-throughput barcoding to analyse community composition, is also resulting in ever-increasing numbers of unidentified fungal ITS sequences [48], a challenge clearly articulated in the recent review by Hibbett *et al*. [17]. The main public repository for these sequences is the Sequence Read Archive (SRA) of NCBI. Current bioinformatic tools and techniques diagnose molecular operational taxonomic units (MOTUs) or species hypotheses from these sequences using similarity thresholds (e.g. 0.03 for ectomycorrhizal (EM) fungi or 0.05 for endophytes). This standardization of unit diagnosis using sequence data allows fungal ecologists to compare across studies and geographical areas. Although sequence clusters are potentially uncoupled from other biologically meaningful information and may not always correspond to recognized species [49–51], it is clear that closely related fungi can be detected repeatedly with this approach, enabling diversity comparisons to be made.

The outputs of conventional specimen-based barcoding and community sequencing thus share the common problem of linking sequences (and sequence clusters) to names. One approach to tackle this is akin to that of the barcode identification number system used in animal barcoding [52]. This involves the establishment of a separate nomenclature based around sequence clusters; this approach is used in the UNITE database [36]. This sequence cluster framework can then be mapped to existing taxonomic infrastructure where sequence clusters/MOTUs overlap with named specimens. The alternative approach, advocated by Hibbett and co-workers [17,53], is for mycologists to collectively work to alter common practice in applying the International Code of Nomenclature, allowing species to be formally named with binomials based on sequence types alone or based on environmental samples.

Regardless of the mechanism, the importance of using well-curated fungal collections as a source of expert taxonomic opinion and authoritative-type material cannot be overstated, and can serve to integrate new sequence data with taxonomy and its important legacy of associated biological and evolutionary knowledge [54,55]. Although ITS has been successfully sequenced from fungus-type material over 200 years old [56,57], this is the exception. Shotgun sequencing of historic material [58] may represent a new opportunity to access genetic information in historical specimens, potentially revolutionizing our ability to stabilize nomenclature and improve connections between sequences, specimens and names. It is clear that both integrating retrospective data from existing collections and routinely sequencing new collections (including generating sequences from all new species) are needed.

## 4. Barcoding successes

An important success of fungal ITS barcoding and the tools devoted to its use (e.g. table 1) is the increased ability to include fungi in studies of biological diversity. Although few fungal researchers use the term barcoding, ITS sequencing is now often a routine part of diversity assessment, particularly for unexplored habitats and regions. Because most of the world's fungi have not been detected by traditional surveys, basic distributional data about the species diversity for most geographical regions and fungal groups are often lacking [59].

### (a) Location-based insights into diversity

At the level of a local assemblage, barcoding approaches have relatively rarely been carried out on individual specimens, because most fungi are usually microscopic. However, lichens and EM fungi produce perennial structures and can provide tests of the method. In lichens, a floristic specimen-based barcoding approach identified a larger proportion of specimens than taxonomists owing to its greater ability to identify scanty, immature and poorly developed material [60]. This does depend on having a reference database available, and in the less well-known lower latitudes, the lack of suitable sequenced reference material for comparison still acts as a constraint [61]. For EM fungi, barcoding of root tips shows greater diversity at sites than above-ground identification of fruiting bodies, even with up to 50 years of fruit–body surveys [11,51,62,63].

Metabarcoding studies have been on scales ranging from the tiny (e.g. the size of insect guts [64] and leaves [65]) to whole-forest soils [48]. In most such studies, the two-stage process of species discrimination and species identification entirely relies on bioinformatics pipelines to streamline analysis of many thousands of newly generated sequences (see section Fungal barcoding databases). Not surprisingly, using this approach, the number of new species clusters discovered by ITS has been growing relative to specimen-based sequences, with little overlap between taxa found in specimen-based compared with environmental samples [17], and total estimates of diversity limited by the inability of studies to reach saturation in rarefaction curves [1,48,66]. For instance, endophyte diversity using ITS sequence data typically far exceeds that found using morphotypes (even with a conservative sequence similarity of 95%; [67]).

### (b) Taxon-based studies

Because of the cryptic nature of the fungal lifecycle, a large degree of unseen diversity is expected. This exists across taxonomic ranks, with new class and even phylum-level divergences being documented, with a particularly rapid expansion in known fungal diversity stemming from sequencing of environmental samples [68,69].

Although multilocus sequencing is likely to remain the gold standard for the unambiguous definition of new species [70–72], data from ITS have been a steady component of fungal diversity description since the early 2000s. Numerous examples of cryptic species have been described, with unrecognized genetic diversity hidden in what was assumed to be a single lineage, e.g. [73], even from quite small sampling areas (e.g. 400 m^2^, [74]). Biologists have long been aware of cryptic species [75], with perhaps the most extreme example from a single basidiolichen now known to represent at least 126 species based on ITS divergence, each with a recognizable combination of traits, including morphology, habitat and distribution. Hundreds more species belonging to this morphology were predicted from unsampled geographical areas [76]. Other lichens and form genera in asexual fungi offer similar cases of extreme polyphyly hidden by seemingly similar morphologies [77,78].

At larger spatial scales, one repeated finding is that fungal taxa with wide distributions are likely to comprise different and isolated genetic lineages sharing exceedingly similar morphology [79–81]. Often, names based on first-described types have been applied to similar morphologies as the nearest approximation of a species hypothesis in another geographical area [82,83], and fungal species with broad geographical distributions are likely to represent fertile areas for discovery of cryptic species [84,85]. The one caveat to this is the trend of widespread high-latitude distributions for many fungal taxa across the arctic [86–88]. A key practical issue in unravelling the complexity of widespread named taxa is effective sampling. Low-intensity sampling from a restricted part of the distribution may generate apparently distinct sequence clusters that then merge as further sampling across the range is undertaken [89].

### (c) Ecology and biogeography

Although a succession of individual species-based studies have shown that fungi are distributed in biogeographically distinct patterns [90], the availability of large datasets from high-throughput studies means that global trends can begin to be examined for fungi in a meaningful way. Ongoing debates about primer choice notwithstanding (electronic supplementary material), the rapidly accumulating findings are at last opening a window into biogeography and diversity for unseen, uncultured and uncollected fungi [59]. For both endophytes and soil fungi, the general trend of species diversity increasing with decreasing latitude has been supported by ITS data [65,91,92]. In contrast, EM fungi appear to be more diverse in the temperate zone [92–95], corresponding to general trends of high Basidiomycota diversity in temperate *Fagus* [96] and pine [1] forests. Similarly, in a global sample of indoor air, latitude was the best predictor of fungal diversity rather than the details of the buildings sampled, and temperate diversity was higher than tropical [97]. Analyses across the arctic have shown no decline in EM fungal diversity from two host plants with increasing latitude [98], and increasing dominance of Ascomycota, including the majority of lichens [99].

Metabarcoding studies of fungi have shown similar biogeographic patterns to other organisms [100], but have also revealed a surprising level of local distributions, potentially sensitive enough for determination of geographical origins of dust for forensic or archaeological application [101]. Supporting the idea that fungal endemism is widespread, metadata mined from unidentified fungal ITS sequences in the INSDC databases allowed a comparison across EM genera, showing that a small handful of poorly known genera such as *Inocybe*, *Tomentella*, *Cortinarius* and *Russula* are often encountered, with high numbers of unidentified sequences (e.g. widespread genera, but not widespread species) [102]. In one example, 0–40% of sequences of *Inocybe* were identified to the species level, depending on their continent of origin, with lower numbers of identified sequences come from Asia and Australia, where reference material is poorly represented in databases [102] and where tropical regions have higher degrees of endemism [92]. However, there are exceptions, and some species were apparently widespread with over 35% of species found on more than one continent [102]. A meta-analysis of published ITS sequences from the truffle genus *Tuber* documented 126 ITS phylotypes, with none sharing intercontinental distributions [103].

One of the best-studied groups of fungi, the EM plant associates, has been used to address the long-standing question about how the diversity of fungi is associated with the diversity of plants. Although there is a general geographical bias in studies of EM fungi favouring Western Europe and North America, in a review of 100 studies, EM fungal diversity was shown to be better explained by host-plant genera than by plant species or family-level diversity [104]. Even some of the best examples of highly specific plant–fungus symbioses have associations that link fungal species groups to host-plant genera [105,106]. Similarly, in fungal–algal associations in lichens, extreme host specificity at the species or strain level tends to be the exception rather than the rule [107]. Typical patterns demonstrate the specificity of fungi for their algal hosts above the species level [108–111]. Likewise, in a global meta-analysis of arbuscular mycorrhizal fungi in the Glomeromycota, fungal community differences are related to geographical distance, climate and plant community [112].

### (d) Conservation applications

Although fungi are often poorly represented in conservation plans compared with plants and animals, they are of considerable conservation relevance. Fungal species can act as bioindicators of habitat status and type, and indicate sites with long ecological continuity [113]. Fungi are also involved in a myriad of complex, often unseen, interactions that are crucial in the functioning of many ecosystems. Individual species can also provide societal benefits in terms of nutrition, medicine, aesthetics and/or cultural values, and hence warrant conservation in their own right. Given that DNA barcoding can improve species discovery and an understanding of fungal distributions [114,115], it can, by extension, improve conservation decision-making.

Metabarcoding datasets have been compared with specimen-based inventory data for invertebrates and birds, and have shown general comparability in relative assessments of alpha and beta diversity, in addition to having the advantages of being much more efficient in terms of person-hours, and amenable to audit by third parties [116]. Such approaches have great potential to understand diversity, distributions and trends in fungi to inform conservation policy and practice. However, one challenge is the potential for disengagement of conservation agencies (e.g. conservation non-governmental organizations) and natural history societies whose working ethos is based around named species and whose efforts have been critical to establishing data on fungal diversity and distribution studies to date. Thus, effective systems to connect sequence data to the existing taxonomic framework are important from a conservation perspective [117] for maintaining cultural connections to fungal diversity. Convenient but non-Linnean names (e.g. soil clone group 1, [69]) can represent a barrier to uptake by land managers, local agencies and decision-makers in many countries.

### (e) Wider societal applications

The practical applications of insights from barcoding may be profound for natural and human systems. Detection of plant pathogens has huge economic implications for both forestry [118] and crop plant systems, where a single pathogen can potentially impact a crop worth billions [119]. At tactical timescales, the detection of cryptic species is crucial to understand major ecological change in European woodlands: divergent ITS types distinguished *Hymenoscyphus fraxineus*, the novel disease agent causing ash dieback [120], with the increasingly apparent impact in the UK [121]. From a human-health perspective, barcoding can extend to indoor mycology [97,122,123] and the importance of fungi contributing to both health and illness in the human microbiome [124], in addition to the obvious application to identification of human and animal pathogens [6], for which diagnostic inaccuracy represents a serious shortcoming [125]. Barcoding approaches are also applicable to industry, in food traceability and understanding industrial composting processes [126–128].

## 5. Where ITS barcoding fails

It is estimated that barcoding using the ITS amplicon is effective for species discrimination across more than 70% of fungi tested [10]. For a barcoding approach to be successful, the variation between species should exceed that within species, with barcodes from a given species best matching conspecifics. The use of ITS sequences for species diagnosis was questioned early on when divergent ITS2 sequences were detected in *Fusarium* [129], and although uncommonly reported, they appear to be taxonomically widespread [130–132] and sometimes linked to hybridization [133]. Intragenomic heterogeneity in ITS may be more prevalent than is currently appreciated, found in several unrelated ascomycete and basidiomycete genera [134,135]. In Glomeromycota, species are multinucleate with extreme intraspecies divergence in nuclear ribosomal sequences, which creates additional challenges for the use of ITS for species discrimination [136].

On the other hand, the lack of sufficient ITS variability has also been a problem, especially in Ascomycota. In some species-rich genera, ITS amplicons that are shorter than the 500 bp recommended for an effective barcode marker are typical [137], resulting in many species having insufficient variation to discriminate important biologically significant groups or closely related species [23]. Although the ITS cistron can correctly identify fungi to the genus level, species discrimination is poor for many plant pathogenic fungi in economically important genera such as *Alternaria*, *Diaporthe*, *Fusarium*, *Teratosphaeria* and others [23].

## 6. Secondary barcode markers

In some lineages, protein-coding genes may have equal or better resolving power than ITS, although these suffer from the lack of universal primers and unreliable amplification [10,138]. In some economically important fungi, genus-specific techniques have been developed which sometimes incorporate ITS along with other markers in multilocus sequence typing [139,140]. This can be used for sequence matching and strain identification [31]. Efforts are underway to propose protein-coding secondary barcodes for specific groups of fungi [141–143]. Additionally, the broad application of using protein coding markers to directly sample environments has been demonstrated recently [144]. This suggests that improved amplification methods may allow for protein-coding genes to act as near-universal DNA barcodes and it will be worthwhile to consider an expansion beyond ITS alone as the barcode marker.

## 7. Integrating DNA barcoding with genomic studies

Fungal genomes provide robust scaffolds that can improve phylogenetic resolution [145], and phylogenomic analyses have proved key for understanding evolutionary relationships in some fungal groups, such as yeasts [146,147]. Sequencing costs continue to drop, new technologies promise rapid and portable platforms that increase the accessibility of genomic sequencing (e.g. Oxford Nanopore's MinION), and ambitious efforts to compile large-scale genome-level data have been proceeding, such as the 1000 fungal genomes [148] and the Plant and Fungal Tree of Life [149]. Already, over 2000 fungal genome projects are underway or complete [150]. However, these initiatives still require considerably more research investment in data gathering and data analysis. For example, multispecies coalescent approaches sacrifice scalability and efficiency in addition to computational time for multilocus versus single-locus approaches [151], and phylogenomic analysis is complicated by gene tree incongruence and the increased sensitivity to long branch attraction from concatenated alignments [146,152]. However, these problems are surmountable, and many nuclear phylogenomic datasets confirm current phylogenetic hypotheses [153].

Although it seems unlikely that whole genome comparisons will displace ITS-based barcoding for fungi in the near future owing to consumables costs and especially the degree of bioinformatics expertise required, there are already several approaches to compare whole genomes that would mirror a DNA-barcoding approach without the need for full-scale phylogenomics. A sizable percentage of known bacterial species have multiple genomes deposited at GenBank. This includes genome data obtained from type cultures for close to 30% of all bacterial species. The use of average nucleotide identity (ANI) and kmer score comparisons are feasible for fast identification of misidentified bacterial genomes [47,154]. Although eukaryotic genomes certainly pose more complex challenges, some of the bacterial approaches could be scalable to fungi [155]. It seems likely that the yeasts will be the first lineages of fungi where this will become a reality in the near future [156]. An important step in this process is linking standard ITS barcoding with genome sequencing projects. Public genome assemblies frequently do not include sequences from nuclear ribosomal RNA cistron, and when they are included, it is often as incorrect or low-quality assemblies. A simple practical step to promote future comparability of fungal datasets is to increase efforts in providing reliable ribosomal data for samples that have their genomes sequenced.

## 8. Conclusion

Fungal research has benefited tremendously from DNA-barcoding approaches and the growing collection of sequences in public, curated databases. Applications range from critical identifications of pathogens to global-scale investigations of fungal diversity. However, the scale of the challenge posed by the sheer diversity of fungi is enormous. Pooling resources to identify and tackle knowledge gaps is therefore essential, and the mycological community has already actively promoted several large-scale collaborations [23,157,158].

The world's preserved fungal collections in herbaria represent an underused resource for building up voucher-based reference datasets [20]: collections-based sequencing is an important priority for the coming decades. Likewise, another step of key importance is to increase the proportion of newly described species that have barcode sequences from the type material. Nevertheless, it is also clear that most fungal diversity will remain uncollected and uncultured, and for the foreseeable future will be known only from environmental samples and sequences. There is thus an urgent need for the fungal research community to unite behind a common approach linking sequences to an effective, scalable method of naming. This approach needs to maximize linkages between ITS barcode sequences and the existing taxonomic framework encompassing specimens, morphological taxonomic descriptions and species concepts. It also needs to encompass the growing depth of sequence coverage given the inevitable increase in genome-level sequencing and the need for multilocus data to provide species-level resolution in many fungal groups.

## Supplementary Material

Supplementary Information
