# Supplementary material for: Scaling up discovery of hidden diversity in fungi: impacts of barcoding approaches
Source: Philos Trans R Soc Lond B Biol Sci. 2016 Sep 5;371(1702):20150336. doi: 10.1098/rstb.2015.0336 (PMC4971188; doi:10.1098/rstb.2015.0336)
Supplement: Supplementary Information [file rstb20150336supp1.pdf]

Yahr R, Schoch CL and Dentinger BTM. 2016. Scaling up discovery of hidden diversity in fungi: impacts of barcoding approaches. *Phil. Trans. R. Soc. B* **371** doi: 10.1098/rstb.2015.0336

Supplementary Information

Contents

1. Fungal Barcoding Resources Supplement P 1.
2. Data for Figures P 5.

1. Fungal Barcoding Resources Supplement

Extraction methods for improving yields and efficiency (Dentinger *et al.* 2009; Osmundson *et al.* 2013a) have been suggested, and to ensure high quality and representativeness, methodological improvements like using proofreading polymerase (Oliver *et al.* 2015), testing primers for bias against certain taxonomic groups (Bellemain *et al.* 2010; De Beeck *et al.* 2014; Tedersoo *et al.* 2015), and even user's guides to 96-well specimen-based (Eberhardt 2012) and high-throughput fungal amplicon sequencing have been published, with step by step suggestions and cautions from sampling and lab methods to analysis and interpretation (Lindahl *et al.* 2013). Perhaps the most discussion has been paid to choice of primers, since so-called universal primers for rDNA have known mismatches for several groups of Fungi, some of which are abundant and presumably ecologically important (e.g for ITS; Rosling *et al.* 2011).

Fungal metabarcoding is almost always carried out using part or all of the ITS region, but there is some controversy about which portion and which primers to use to offer the best resolution with the best representativeness (Blaalid *et al.* 2013). The choice has important implications for species identification and any downstream application of those names (Nilsson *et al.* 2008). Some workers have suggested co-amplification with SSU in order to allow for phylogenetic studies and to anchor so-called orphan ITS sequences with no close analogues in databases (O'Brien *et al.* 2005; Richards *et al.* 2012), but as metabarcodes are necessarily limited in length by current sequencing technologies, most arguments are focussed on which part of the ITS should be targeted. If sequence read length is set to continue to increase with new technologies, ITS2 would benefit from the higher resolving power of the downstream LSU compared with ITS1 and its highly-conserved downstream 5.8S (Nilsson *et al.* 2008). On the other hand, ITS1 is reported to be more variable than ITS2 for a majority of basidiomycetes from dried collections tested

(Osmundson *et al.* 2013b) and offers slightly better resolving power across a wide range of ascomycetes from sequence databases (Wang *et al.* 2015). The latter study and others also reported similar species identification success of ITS1 and ITS2 across a wide range of Basidiomycota (Blaalid *et al.* 2013; Wang *et al.* 2015). In tests in the EM fungal genus *Inocybe* (Ryberg *et al.* 2008), and across lichen fungi (Kelly *et al.* 2011), ITS1 and ITS2 performed more or less equally well at species discrimination, which is not surprising, since ITS1 and ITS variation tend to be correlated (Nilsson *et al.* 2008; Blaalid *et al.* 2013).

The choice of PCR primers has important consequences for what sequences are recovered, with some groups severely underrepresented (e.g. Bellemain *et al.* 2010; Schadt & Rosling 2015), and in some cases yield remarkably low species-level resolution, with only 45% (Pitkäranta *et al.* 2008) or even fewer than 25% of OTUs identified (Korpelainen *et al.* 2015) for indoor air fungi. Group I introns in the SSU can also result in non-amplification or overly long amplicons for some lichens (Kelly *et al.* 2011). Several new sets of primers have been proposed and tested (Toju *et al.* 2012; De Beeck *et al.* 2014; Tedersoo *et al.* 2015). Despite eliminating some taxon bias, there are disadvantages to using newly designed primers, notably the loss of comparability with other studies, and particularly with the difficulty in relative quantification of OTUs. However, to assay the bias in primers, a test in soil fungi found that shotgun sequencing versus amplicon sequencing revealed little to no bias (Tedersoo *et al.* 2015). Further similar tests should be completed in other fungal target groups and habitats.

Several Fungal-specific bioinformatics pipelines have also been developed, the best-known of which is UNITE, which includes the PlutoF workbench (Abarenkov *et al.* 2010) and modules for ITS extraction, chimera checking (including UCHIME(Edgar *et al.* 2011)) and identification, by matching query sequences with species hypotheses (including varying similarity cut-offs) and reference sequences determined by expert users. The intergrated pipeline PIPITS takes advantage of many of the features of UNITE (Gweon *et al.* 2015), and another expressly for Illumina data was created to be both flexible and straightforward, having been used successfully by inexperienced students with only a few hours tuition (Seifert *et al.* 2007). The bioinformatics tools outlined here here are based on MOTU discrimination and similarity thresholds, whereas evolutionary-aware approaches such as phylogenetic and coalescent-based criteria remain marginal and largely restricted to fungal taxonomists. The available fungal metabarcoding workflows are also designed for amplicon sequencing

studies, and to our knowledge there are no optimised approaches for phylogenetic profiling of fungi in shotgun metagenomics datasets like there are for prokaryotes (Segata et al. 2012).

References

Abarenkov K., Tedersoo L., Nilsson R.H., Vellak K., Saar I., Veldre V., Parmasto E., Prous M., Aan A. & Ots M. (2010). PlutoF—a web based workbench for ecological and taxonomic research, with an online implementation for fungal ITS sequences. *Evol. Bioinform. Online*, 6, 189.

Bellemain E., Carlsen T., Brochmann C., Coissac E., Taberlet P. & Kauserud H. (2010). ITS as an environmental DNA barcode for fungi: an in silico approach reveals potential PCR biases. *BMC Microbiol.*, 10, 189.

Blaalid R., Kumar S., Nilsson R.H., Abarenkov K., Kirk P. & Kauserud H. (2013). ITS1 versus ITS2 as DNA metabarcodes for fungi. *Mol. Ecol. Res.*, 13, 218-224.

De Beeck M.O., Lievens B., Busschaert P., Declerck S., Vangronsveld J. & Colpaert J.V. (2014). Comparison and validation of some ITS primer pairs useful for fungal metabarcoding studies. *PloS ONE*, 9.

Dentinger B.T.M., Margaritescu S. & Moncalvo J.-M. (2009). Rapid and reliable high-throughput methods of DNA extraction for use in barcoding and molecular systematics of mushrooms. *Mol. Ecol. Res.*, 10, 628-633.

Eberhardt U. (2012). Methods for DNA Barcoding of Fungi. In: *DNA Barcodes* (eds. Kress WJ & Erickson DL). Humana Press, pp. 183-205.

Edgar R.C., Haas B.J., Clemente J.C., Quince C. & Knight R. (2011). UCHIME improves sensitivity and speed of chimera detection. *Bioinformatics*, 27, 2194-2200.

Gweon H.S., Oliver A., Taylor J., Booth T., Gibbs M., Read D.S., Griffiths R.I. & Schonrogge K. (2015). PIPITS: an automated pipeline for analyses of fungal internal transcribed spacer sequences from the Illumina sequencing platform. *Method. Ecol. Evol.*

Kelly L.J., Hollingsworth P.M., Coppins B.J., Ellis C.J., Harrold P., Tosh J. & Yahr R. (2011). DNA barcoding of lichenized fungi demonstrates high identification success in a floristic context. *New Phytol.*, 191, 288-300.

Korpelainen H., Pietiläinen M. & Huotari T. (2015). Effective detection of indoor fungi by metabarcoding. *Ann. Microbiol.*, 1-4.

Lindahl B.D., Nilsson R.H., Tedersoo L., Abarenkov K., Carlsen T., Kjøller R., Kõljalg U., Pennanen T., Rosendahl S. & Stenlid J. (2013). Fungal community analysis by high-throughput sequencing of amplified markers–a user's guide. *New Phytol.*, 199, 288-299.

Nilsson R.H., Kristiansson E., Ryberg M., Hallenberg N. & Larsson K.-H. (2008). Intraspecific ITS variability in the kingdom Fungi as expressed in the international sequence databases and its implications for molecular species identification. *Evol. Bioinform. Online*, 4, 193.

O'Brien H., Parrent J., Jackson J., Moncalvo J. & Vilgalys R. (2005). Fungal community analysis by large-scale sequencing of environmental samples *Appl. Environ. Microbiol.*, 71, 5544-5550.

Oliver A.K., Brown S.P., Callaham M.A. & Jumpponen A. (2015). Polymerase matters: non-proofreading enzymes inflate fungal community richness estimates by up to 15%. *Fungal Ecol.*, 15, 86-89.

Osmundson T.W., Eyre C.A., Hayden K.M., Dhillon J. & Garbelotto M.M. (2013a). Back to basics: an evaluation of NaOH and alternative rapid DNA extraction protocols for DNA barcoding, genotyping, and disease diagnostics from fungal and oomycete samples. *Mol. Ecol. Res.*, 13, 66-74.

Osmundson T.W., Robert V.A., Schoch C.L., Baker L.J., Smith A., Robich G., Mizzan L. & Garbelotto M.M. (2013b). Filling gaps in biodiversity knowledge for macrofungi: contributions and assessment of an herbarium collection dna barcode sequencing project. *PloS ONE*.

Pitkäranta M., Meklin T., Hyvärinen A., Paulin L., Auvinen P., Nevalainen A. & Rintala H. (2008). Analysis of fungal flora in indoor dust by ribosomal DNA sequence analysis, quantitative PCR, and culture. *Appl. Environ. Microbiol.*, 74, 233-244.

Richards T.A., Jones M.D., Leonard G. & Bass D. (2012). Marine fungi: their ecology and molecular diversity. *Annu. Rev. Mar. Sci.*, 4, 495-522.

Rosling A., Cox F., Cruz-Martinez K., Ihrmark K., Grelet G.-A., Lindahl B.D., Menkis A. & James T.Y. (2011). Archaeorhizomycetes: unearthing an ancient class of ubiquitous soil fungi. *Science*, 333, 876-879.

Ryberg M., Nilsson R.H., Kristiansson E., Töpel M., Jacobsson S. & Larsson E. (2008). Mining metadata from unidentified ITS sequences in GenBank: a case study in *Inocybe* (Basidiomycota). *BMC Evol. Biol.*, 8, 50.

Schadt C.W. & Rosling A. (2015). Comment on "Global diversity and geography of soil fungi". *Science*, 348, 1438.

Seifert K.A., Samson R.A., Houbraken J., Lévesque C.A., Moncalvo J.-M., Louis-Seize G. & Hebert P.D. (2007). Prospects for fungus identification using CO1 DNA barcodes, with Penicillium as a test case. *Proc. Nat. Acad. Sci. U.S.A.*, 104, 3901-3906.

Tedersoo L., Anslan S., Bahram M., Polme S., Riit T., Liiv I., Koljalg U., Kisand V., Nilsson R.H., Hildebrand F., Bork P. & Abarenkov K. (2015). Shotgun metagenomes and multiple primer pair-barcode combinations of amplicons reveal biases in metabarcoding analyses of fungi. *Mycokeys*, 1-43.

Toju H., Tanabe A.S., Yamamoto S. & Sato H. (2012). High-coverage ITS primers for the DNA-based identification of ascomycetes and basidiomycetes in environmental samples.

Wang X.-C., Liu C., Huang L., Bengtsson-Palme J., Chen H., Zhang J.-H., Cai D. & Li J.-Q. (2015). ITS1: a DNA barcode better than ITS2 in eukaryotes? *Mol. Ecol. Res.*, 15, 573-586.

2. Data for Figures.

Figure 1.

Year	Species with unspecified names	Species with binomials
1993	12	438
1994	8	209
1995	31	338
1996	22	450
1997	92	712
1998	266	895
1999	361	1250
2000	624	1427
2001	1106	1422
2002	1586	1724
2003	1640	1463
2004	1656	1589
2005	2184	1316
2006	3430	1809
2007	3797	1436
2008	5978	1968
2009	5460	1537
2010	7132	1908
2011	8287	2086
2012	8928	2272
2013	9689	1910
2014	11307	2289

In order to compare the annual set of binomials and unspecified names the following two sets of queries were done in Entrez Direct for each year since the inception of NCBI Taxonomy in 1993 e.g.:
esearch -db taxonomy -query "Fungi[organism] AND species[rank] AND specified[prop] AND 1993[edat]"
esearch -db taxonomy -query "Fungi[organism] AND species[rank] NOT specified[prop] AND 1993[edat]"

Figure 2.

Year of Publication	New Species in Index Fungorum / MycoBank	Total Binomials in GenBank	%	ITS record in UNITE	%
1990	1210	198	16.364	130	10.744
1991	974	144	14.784	88	9.035
1992	889	198	22.272	125	14.061
1993	1286	193	15.008	128	9.953
1994	1197	217	18.129	115	9.607
1995	1221	226	18.509	147	12.039
1996	1327	268	20.196	179	13.489
1997	1435	245	17.073	162	11.289
1998	1164	292	25.086	181	15.550
1999	1200	278	23.167	167	13.917
2000	1180	286	24.237	189	16.017
2001	1290	375	29.07	278	21.550
2002	1133	251	22.154	166	14.651
2003	1311	332	25.324	229	17.468
2004	1425	504	35.368	362	25.404
2005	968	367	37.913	230	23.760
2006	1161	422	36.348	308	26.529
2007	1424	481	33.778	356	25.000
2008	1423	470	33.029	344	24.174
2009	1599	545	34.084	401	25.078
2010	1270	547	43.071	386	30.394
2011	1516	723	47.691	525	34.631
2012	1772	842	47.517	648	36.569
2013	1802	888	49.279	715	39.678
2014	1981	1080	54.518		

The yearly species names released in Index Fungorum and MycoBank was obtained. Each year's combined set of names was then searched against the NCBI Taxonomy using the Taxonomy name/id Status Report tool (http://www.ncbi.nlm.nih.gov/Taxonomy/TaxIdentifier/tax_identifier.cgi) at NCBI. The output indicated names which were present in the NCBI Taxonomy and each year was subsequently tabulated. The validly published species names in Index Fungorum and MycoBank were obtained for each year. The combined non-redundant set of names was then searched against the NCBI Taxonomy database using the Taxonomy name/id Status Report tool (http://www.ncbi.nlm.nih.gov/Taxonomy/TaxIdentifier/tax_identifier.cgi) at NCBI. The output indicated names which were present/absent in the NCBI Taxonomy. In order to verify how many of these names were associated with ITS records present at the UNITE database we did an Entrez query (species name[orgn] AND loprovunite [filter]) in the Nucleotide Database at NCBI. Subsequently the presences of names in each database were counted and tabulated by the year a new name became published. Note, link out providers such as UNITE maintain their own LinkOut files and the search term loprovunite [filter] is dependent on that. The data for 2014 from UNITE were not up to date at of the date of data retrieval, and therefore not included in the final figure.
